# The research of Tuna Huichun Gong on pulmonary function, exercise tolerance, and quality of life in patients with chronic obstructive pulmonary disease based on the concept of early pulmonary rehabilitation

**DOI:** 10.1097/MD.0000000000020625

**Published:** 2020-06-05

**Authors:** Wei Yu, Peiyuan Su, Jiaojiao Wang, Pengcheng Zhou, Keling Chen, Li Liu, Qianming Xia, Yuewei Chen

**Affiliations:** a Department of Respiratory Medicine, Hospital of Chengdu University of Traditional Chinese Medicine; b Department of Cardiothoracic Surgery, Hospital of Chengdu University of Traditional Chinese Medicine; c Clinical Medical School, Chengdu University of Traditional Chinese Medicine; d Department of Respiratory Medicine, AVIC 363 Hospital, Chengdu, Sichuan Province, PR China.

**Keywords:** chronic obstructive pulmonary disease, pulmonary rehabilitation, randomized controlled trial, Tuna-Hui-Chun-Gong

## Abstract

**Introduction:**

Chronic obstructive pulmonary disease (COPD) is a common high-burden and highly disabling lung disease. The quality of life and exercise endurance of patients with COPD is often low because of atrophy of the respiratory and skeletal muscles. Although recommended by the global initiative for chronic obstructive lung disease guidelines, pulmonary rehabilitation (PR) has not been used widely because of its inherent limitations. Tuna-Hui-Chun-Gong (TNHCG) is a popular traditional exercise used to treat COPD in China. We aim to evaluate the safety and efficacy of TNHCG for PR of COPD.

**Methods:**

The provided protocol is for a single-blind randomized controlled trial in which 120 COPD patients will be randomly and equally divided into the experimental or control group. The control group will be treated with standard COPD drugs while the experimental group will perform TNHCG exercises apart from standard drug treatment. The duration of treatment will be 24 weeks and a follow-up for 48 weeks. The primary outcome will be the 6-Minute Walk Test. The secondary outcomes will include the pulmonary function test, St George's respiratory questionnaire, COPD assessment test, modified medical research council dyspnea scale, Hospital Anxiety and Depression Scale, and exacerbation frequency. A safety assessment will also be performed during the trial.

**Discussion:**

Our study will provide evidence to support TNHCG exercise as an additional measure for PR of COPD.

**Trial registration:**

ChiCTR1900028332, Registered December 29, 2019.

**Ethics and dissemination:**

Ethics approval has been granted by the Sichuan Traditional Chinese Medicine Regional Ethics Review Committee (No. 2019KL-050).

## 1 Introduction

Chronic obstructive pulmonary disease (COPD) is a chronic disease characterized by shortness of breath, chronic cough, and poor exercise tolerance.^[1]^ Although the symptoms of COPD can be well controlled with drugs, patients often end up having physiological dysfunctions and lower quality of life due to respiratory and skeletal muscle fatigue and atrophy. Consequently, pulmonary rehabilitation (PR) for COPD has recently gained importance. Numerous studies have confirmed that PR not only improves dyspnea, health status, quality of life, and exercise tolerance in patients with COPD, but also reduces hospitalization time for patients who experienced exacerbation and symptoms of anxiety and depression.^[2–4]^ Moreover, PR has been recommended in various versions of the global initiative for chronic obstructive lung disease (GOLD) guidelines.^[5]^ According to the definitions proposed by the American Thoracic Society and European Respiratory Society, PR refers to interventions that are performed after a comprehensive assessment of patients, and include education, sports training, nutritional support, and behavior change, with exercise being a key component of PR.^[6]^ The goal of PR is to improve the physical and psychological conditions of patients with chronic respiratory diseases and promote their long-term compliance with a healthy lifestyle. Previous studies have confirmed that traditional sport exercises such as Tai Chi, Yoga, Yi Jin Jing, and Wu Qin Xi promote PR of COPD patients and improve their lung function and quality of life.^[7–10]^ To date, however, there has been no high-quality clinical trial that assessed the effectiveness and safety of Tuna-Hui-Chun-Gong (TNHCG) exercise for rehabilitation of COPD.

TNHCG is mainly composed of Wudang, which originated from a Taoist health care sport named WuxingGong. It includes tortoise-shaped postures to help improve balance, posture alignment, weight shifting, circular movements, relaxation, and breath control. Compared to other exercises, tortoise-shaped posture exercises have simple moves and focus on breath control. Some studies showed that tortoise-shaped posture exercises improve 6-Minute Walk Test (6-MWT) scores, lung function, and COPD Assessment Test (CAT) score during the stable period of COPD.^[11–13]^ Apart from the tortoise-shaped posture exercises, TNHCG also includes lip contraction breathing, abdominal breathing, and acupressure, all of which improve PR for COPD as confirmed by previous studies.^[14–16]^ In our hospital, TNHCG has been widely used in PR of chronic lung diseases for many years. Clinical observation revealed that it improves symptoms, quality of life, and exercise endurance of patients with chronic lung diseases. However, whether it also plays an important role in lung rehabilitation of COPD is not clear. Nevertheless, it is reasonable to hypothesize that TNHCG will be effective for the rehabilitation of COPD. Therefore, we designed this randomized controlled trial to evaluate the efficacy and safety of TNHCG for the rehabilitation of COPD.

## 2 Methods and design

### 2.1 Design

**Figure 1:**
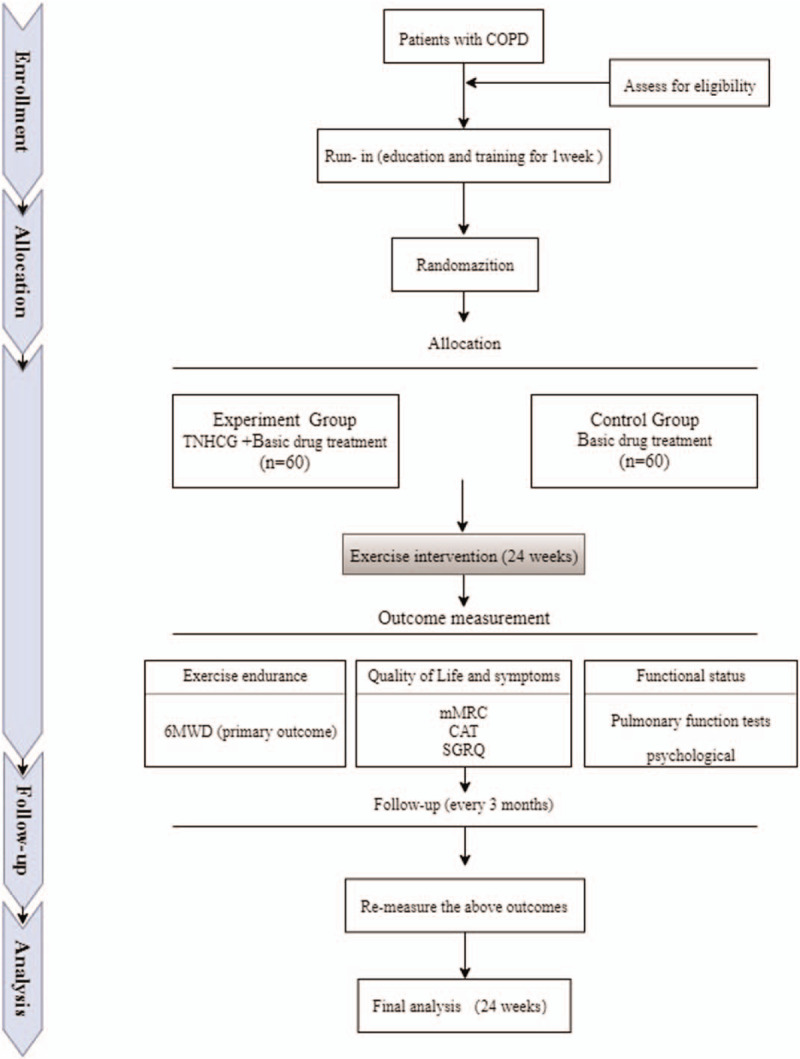
Flow chart of the study design. 6MWT = 6-Minute Walk Test, CAT = COPD assessment test, mMRC = The Modified Medical Research Council Dyspnea Scale, SGRQ = St George's Respiratory Questionnaire.

**Figure 2:**
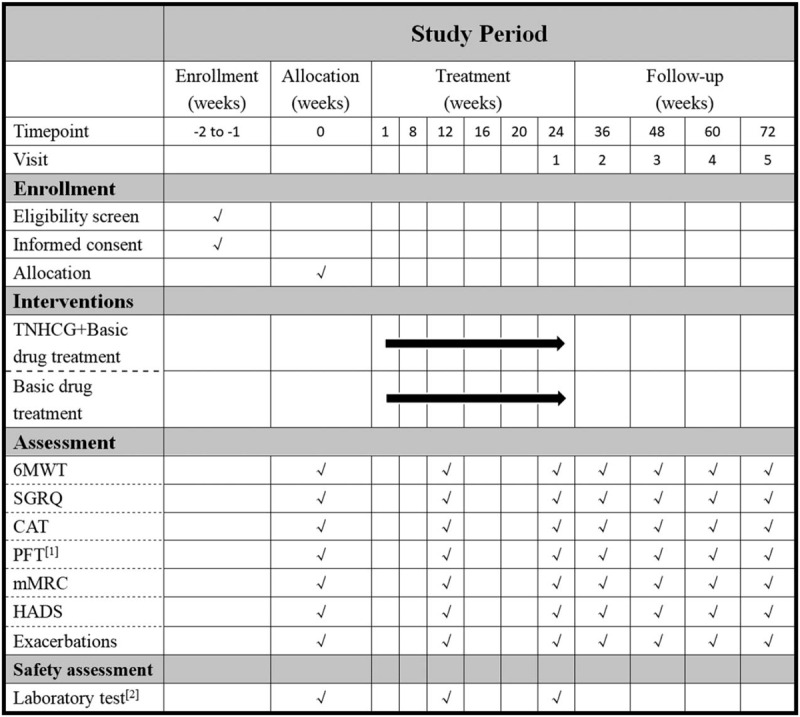
SPIRIT figure: Schedule of enrollments, interventions, and assessments. 6MWT = 6-minute walk test, CAT = COPD assessment test, HADS = The Hospital Anxiety and Depression Scale, mMRC=The Modified Medical Research Council Dyspnea Scale, PFT = Pulmonary function tests, SGRQ = St George's Respiratory Questionnaire, [1] PFT: FEV1, Forced expiratory volume in one second; FVC, Forced volume capacity. [2] Laboratory tests: blood, urine, feces, electrocardiogram, and kidney and liver function tests.

The following is a protocol for a prospective randomized controlled clinical trial. The trial has been registered with the Chinese Clinical Trial Registry (no ChiCTR1900028332, registered December 29, 2019). After obtaining written informed consent, a 1-week run-in period will be implemented, after which 120 eligible participants will be randomly assigned to a trial group or a control group in a 1:1 ratio. The 2 groups will then undergo treatment for 24 weeks with follow-up after 12 weeks. The study procedure is shown in Figure 1. The trial aims to investigate the additional benefits and safety of TNHCG compared to conventional drug treatment for COPD. It will adhere to the Standard Protocol Items: Recommendations for Interventional Trials (SPIRIT) 2013 statement. The schedule of enrollments, interventions, and assessments is shown in Figure 2. The SPIRIT 2013 checklist is presented as Additional File 1.

### 2.2 Ethics approval

The study is in compliance with the Declaration of Helsinki (Edinburgh 2000 version). The final amendments (version: November 4, 2019) and the consent form have been reviewed and approved by the Ethics Committee at Hospital of Chengdu University of Traditional Chinese Medicine (approval no: 2019KL-050). If there are any amendments to the protocol, approval will be sought again from the ethics committee.

### 2.3 Recruitment

Participants will be recruited via an advertisement through media and based on the advice of respiratory doctors at the Chengdu University of Traditional Chinese Medicine hospital (Chengdu, China). Before enrollment, participants will be provided with detailed information about the clinical study, including its purpose, procedure, schedule, and possible risks and benefits. Only those who agree to sign the informed consent form will be included in the study.

### 2.4 Sample size

Sample size calculations are based on the primary outcome (improvement in the 6-MWT). Referring to previous research by Chen et al,^[17]^ compared to the conventional treatment group, the 6-minute walking distance (6MWD) of the COPD TaiChi PR group improved by an average of 52 m (variance 77.5, correlation coefficient 0.7–0.8, power 0.90). Power analysis revealed that 96 participants, 48 in each group are required to attain a significance level of 2.5%. To avoid the decrease in sample size caused by follow-up drop-outs, the total sample size required, calculated with a 20% drop-out rate, is 120 participants with 60 participants per group.

### 2.5 Randomization and blinding

A professional statistician with expertise in evidence-based traditional Chinese medicine in the Sichuan province will generate 120 random serial numbers using SAS 9.2 software (SAS, Cary, NC). Randomization will be performed after screening and baseline assessment, and eligible patients with COPD will be assigned to the trial group or the control group in a 1:1 ratio. The allocation sequence will be kept in sequentially numbered, sealed, and opaque envelopes. The envelopes will be kept safe and opened only by a designated administrator who will not directly participate in the recruitment or follow-up of any participant. The main investigator, data manager, data analyst, and outcome evaluators will be blinded to the group allocations. Participants and TNHCG instructors who supervise the exercise will not be blinded to the allocation. Once the groups are allotted, the TNHCG instructors will receive a copy of the participant number and allocation, and the outcome assessor will be informed of the participant number only.

### 2.6 Diagnostic criteria

Participants must meet the diagnostic criteria mentioned in the GOLD guidelines^[5]^ (Table 1).

**Table 1:**
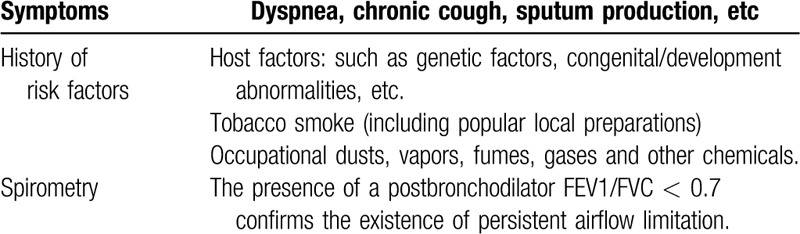
Diagnostic criteria for chronic obstructive pulmonary disease^[5]^.

### 2.7 Eligibility criteria

Inclusion criteria:

1. Patients aged 40 to 75 must meet the diagnostic criteria of COPD according to the GOLD guidelines; no gender and grade limitations.
2. Patients with acute exacerbation periods must first be relieved before they can participate in the trial, while patients with stable COPD meet the inclusion criteria.
3. Patients have no heart, liver, kidney, or mental system issues.
4. Patients provide written informed consent.

Exclusion criteria:

1. Participants with other chronic respiratory diseases.
2. Participants with severe comorbidities, such as tumors, symptomatic cardiovascular diseases, myocardial infarction, and neuromuscular diseases.
3. Participants who are pregnant or lactating.
4. Participants with AIDS, mental illnesses, and cognitive dysfunction.
5. Participants who are currently practicing traditional Qigong exercises such as Baduanjin and TaiChi or are participating in other clinical trials associated with COPD.

### 2.8 Termination and withdrawal criteria

All participants will be informed that they have the right to withdraw from the trial at any point and that they will be provided with standardized treatment if they withdraw. The reason for withdrawal will be recorded in their case report file. The criteria for stopping treatment and withdrawing patients from the clinical trial are as follows: the participant developed adverse event due to TNHCG exercise, and the investigator believes it is not appropriate for them to continue the trial; the participant developed severe exacerbation of COPD; the participant developed another severe disease that needs to be treated during the study; poor compliance; the patient participated in another similar trial simultaneously.

## 3 Interventions

### 3.1 Treatment plan

Participants in the 2 groups will receive the same kind of education about COPD. The contents include smoking cessation, nutrition support, inhalation techniques, coughing techniques, and knowledge of COPD.

The control group will only be treated with standard COPD drugs, while the trial group will perform TNHCG exercises apart from standard drug treatment.

**Figure 3:**
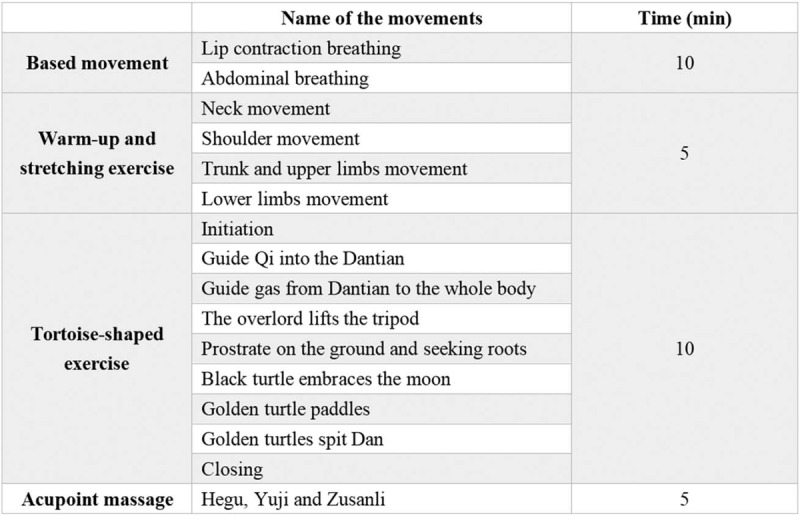
The content of TNHCG includes Based movement, Warm-up and stretching exercise, Tortoise-shaped exercise, and Acupoint massage.

Participants in the trial group will perform TNHCG exercises five times a week, totally 30 minutes per time, for 24 weeks. Before the start of the trial, a designated person (a nurse or rehabilitation physician not involved in conducting this study) will guide the participants on how to perform TNHCG exercises at our hospital. Participants will be required to practice until their movement and breathing are coordinated. If a patient is discharged from the hospital, a designated person will keep in touch with them and continue to guide them through telephone, video, and WeChat every week. The specific exercise content of TNHCG is shown in Figure 3.

### 3.2 Monitoring of exercise intensity

There are numerous methods to monitor the intensity of exercise, such as the patient's heart rate (HR), subjective symptoms, and maximum oxygen uptake. In clinical practice, we often use the patient's target HR and conscious feelings as monitoring indicators. The patient's HR during exercise should be less than the target HR. The formula to calculate target HR is as follows^[18]^: target HR = [(220 - age) - resting HR] ^∗^ %HRR + resting HR. This method requires professional instructors to determine the patient's resting heart rate before exercise. In our trial protocol, participants’ self-perception is used as the monitoring indicator. During exercise, the target exercise intensity is as follows: the patient does not feel tired and sweats slightly throughout; there are no breathing difficulties, joint, or severe muscle pain; an increase in HR of <20 beats per min; respiratory rate <5 breaths per min over resting respiratory rate.

### 3.3 Outcome measures

#### 3.3.1 Primary outcome

##### 3.3.1.1 6-Minute Walking Test

The primary outcome is the change in 6-MWD from baseline to the end of the treatment period (week 24). The purpose of this test is to measure the maximum distance that the participants can walk as fast as possible within 6 minutes on a flat surface. The 6-MWT is one of the most widely used tests to assess exercise tolerance in COPD.^[17]^ Participants will be given 400 μg salbutamol aerosol 20 min before the tests for safety.

#### 3.3.2 Secondary outcomes

##### 3.3.2.1 Pulmonary function test

Pulmonary function is the gold standard parameter for the diagnosis of COPD and is recommended by the GOLD guidelines.^[5]^ The pulmonary function test will be performed by a professional with a designated instrument (JAEGER MasterScreen, CareFusion, Höchberg, Germany). Participants will be administered bronchodilators before the tests. The parameters FEV1, FEV1 predicted, FVC, FVC predicted, and FEV1/FVC will be recorded.

##### 3.3.2.2 St George's Respiratory Questionnaire (SGRQ)

The SGRQ includes 3 parts: symptoms, mobility, and social and emotional effects of the disease, which is an important tool for assessing the quality of life of patients with COPD.^[19]^ Study researchers will guide the participants in filling the questionnaire and participants will be required to answer the questions honestly.

##### 3.3.2.3 Modified Medical Research Council Dyspnea Scale

The Modified Medical Research Council Dyspnea Scale is classified into 5 grades (0–4) based on the severity of dyspnea and can be completed in 30 seconds. Presently, it is widely used for assessment of dyspnea assessment as well.^[20]^

##### 3.3.2.4 COPD Assessment Test

The CAT was presented by Jones based on the SGRQ in 1991. The CAT includes parameters such as symptoms, exercise capacity, psychology, sleep, and impact of society. It is a very important tool for assessing the severity of COPD symptoms.^[19]^

##### 3.3.2.5 Hospital Anxiety and Depression Scale

The Hospital Anxiety and Depression Scale is a self-administered questionnaire used to screen for the presence of depression and anxiety and has been shown to be a valid and reliable measure of severity of emotional disorders used in general practice.^[21]^ Participants will be instructed to choose one response from the given answers that best describes their current feelings.

##### 3.3.2.6 Exacerbation frequency

The number of acute exacerbation episodes of COPD (at week 36). COPD exacerbation is defined as an acute worsening of respiratory symptoms resulting in the requirement of additional therapy.^[22]^

### 3.4 Safety assessment

TNHCG, which is mainly composed of Wudang tortoise-shaped posture exercises has been used for disease treatment, health care, and rehabilitation for more than 2000 years in China. It includes low to medium intensity exercises and previous studies have confirmed that it has a good effect on COPD rehabilitation with few adverse reactions.^[11–13]^ In this study, all procedures will strictly follow the standard operation procedure (SOP) of TNHCG exercise. Moreover, laboratory tests will be performed to analyze blood; urine; feces; and heart, liver, and kidney function; from the time of enrollment through the treatment period.

### 3.5 Compliance

The participants will be informed about test content, course of intervention, and adverse effect of the trial in detail before being allocated to a group. Once patients have been randomized, researchers will make an effort to follow the patient for the duration of the study. At each visit, adherence to interventions will be monitored, the results of all examinations explained, and all trial or transportation costs will be covered. Before every visit, messages will be sent through media such as WeChat, QQ, or by phone to remind patients of the upcoming visit for data collection. Otherwise, free registration and treatment advice will be provided to the participants in the follow-up period.

### 3.6 Adverse events

Any adverse events will be recorded in the CRFs irrespective of their relationship to the study intervention. In case of any serious adverse events, the intervention will be immediately stopped and a detailed description of the time, severity, relationship with TNHCG exercise, and the measures taken based on standard procedures of the China Food and Drug Administration will be recorded. In addition, serious adverse events will be reported to the Steering Committee and Ethics Committee within 24 hours.

### 3.7 Data management and quality control

All data will be recorded in the CRFs, which will be completed by trained and qualified investigators. Once a CRF is completed, the original record will not be changed if any corrections are made. The completed CRFs will be reviewed by the clinical inspector. Data entry and sorting will be performed using EpiData 3.1 software; 2 data administrators will input and proofread the data independently. After reviewing and confirming that the established database is correct, the data will be locked by the main researchers and statistical analysts. All data management will be according to the standard procedures. The evidence-based Medicine Center at the Sichuan TCM hospital (Chengdu, China), which does not have any competing interests, will be responsible for monitoring the data. The Department of Science Research of the hospital at Chengdu University of TCM, which is independent of the investigators, will perform data audits during the trial.

### 3.8 Statistical analysis

All analyses will be conducted according to the intention to treat principle. Missing values will be replaced by the last observation carried forward method. Two similar participants with complete data will be double-checked to make sure that data are correct before analysis.

The data will be analyzed using the Statistical Package for the Social Sciences version 23.0 (SPSS 23.0, Chicago, IL). The analytical methods will be selected according to the distribution characteristics of the data: measurement data will be analyzed using group *t* tests or nonparametric tests, while count data will be analyzed using a Chi-square test or the Fisher exact probability method. Grade data will be analyzed using nonparametric tests. Compared with baseline values, measurement data will be assessed using paired *t* tests or nonparametric tests, and count data will be examined using a nonparametric test. All statistical tests will be bilateral tests and *P*-values <.05 will be considered statistically significant.

## 4 Discussion

In China, COPD is one of the most commonly occurring respiratory diseases. The latest epidemiological survey shows that the incidence rate of COPD for people over 40 years old is as high as 13.7% with a total of nearly 100 million patients.^[23]^ Although PR in COPD has been widely valued, However, conventional PR exercises require professional equipment, places and personnel, which were not fully applied to the clinic. In China, Tai Chi, Ba Duan Jing, Wu Qin Xi and other traditional Chinese sports are widely used in prevention and rehabilitation of chronic respiratory disease, which has the characteristics of simple movement, suitable strength and no need for special equipment, and is especially suitable for the rehabilitation of COPD. Previous studies have confirmed that it can improve dyspnea, exercise endurance and quality of life, and reduce the frequency of acute exacerbations in COPD patients.^[7,8,24]^ This suggests that traditional Chinese sports are good alternatives to conventional PR for COPD.

TNHCG originated from the Wudang tortoise-shaped posture exercise, which is similar to Tai Chi, and is widely used in Taoist health and rehabilitation centers. Although previous studies^[11–13]^ have shown that Wudang tortoise-shaped posture exercises can improve symptoms, 6-MWT score, and quality of life of patients with COPD, there are some inherent design problems associated with them. For example, the randomization method is not rigorous, blinding and allocation is not described appropriately, correct sample calculation method was not mentioned, and AEs were not recorded in detail. All of these are prone to bias. TNHCG is a set of exercises designed by our hospital specifically for lung rehabilitation of patients with COPD. It targets the pathophysiological areas of COPD such as air trapping and dynamic lung inflation, and contains not only Wudang based tortoise-shaped posture exercises, but also includes lip contraction breathing, abdominal breathing, and acupressure. Although TNHCG has been used in our hospital for nearly 10 years, there has been no relevant research to explore its effectiveness and safety in PR of COPD. Consequently, we designed this randomized controlled trial.

This study may also have some limitations. First, since the aim of the study was mainly to clarify the effectiveness and safety of TNHCG, it has only been compared to a control group that received standard drugs, and not to other conventional PR exercise methods. Therefore, it cannot be concluded whether TNHCG exercise is more or less beneficial than other methods and whether combined use will have a better effect. Second, this is a single-center study with a small sample size. Third, due to limited research funding, we cannot study the mechanism by which TNHCG improves PR. Despite these limitations, we believe that this study will be helpful in elucidating the benefits of TNHCG exercise for PR of COPD. In the future, a multicenter randomized controlled trial with a large sample of patients with COPD must be conducted. Moreover, the mechanism of rehabilitation should be researched.

## 5 Others

### 5.1 Trial status

This paper is based on protocol version 2.0 dated 29 December 2019. The clinical study began in Jan, 2020, and the approximate date of completion is December, 2021. Currently, participants are being recruited.

## Acknowledgments

We are grateful to the Department of Sichuan Provincial Cadre Health for funding this study.

## Author contributions

**Conceptualization:** Pengcheng Zhou, Qianming Xia.

**Investigation:** Jiaojiao Wang, Keling Chen, Li Liu

**Supervision:** Qianming Xia, Peiyuan Su.

**Writing – original draft:** Wei Yu, Pengcheng Zhou.

**Writing – review & editing:** Yuewei Chen, Peiyuan Su.
